# Therapeutic Potential of Gramicidin S in the Treatment of Root Canal Infections

**DOI:** 10.3390/ph9030056

**Published:** 2016-09-07

**Authors:** Marina Berditsch, Hannah Lux, Oleg Babii, Sergii Afonin, Anne S. Ulrich

**Affiliations:** 1 Institute of Organic Chemistry, Karlsruhe Institute of Technology (KIT), Fritz-Haber-Weg 6, Karlsruhe 76131, Germany; marina.berditsch@kit.edu (M.B.); luxhannah89@gmail.com (H.L.); oleg.babii@kit.edu (O.B.); 2 Institute of Biological Interfaces (IBG-2), KIT, P. O. Box 3640, Karlsruhe 76021, Germany; sergii.afonin@kit.edu

**Keywords:** gramicidin S, *Enterococcus faecalis*, tetracycline resistance, biofilms, root canal infections, alarmone ppGpp, polymyxin B

## Abstract

An intrinsic clindamycin-resistant *Enterococcus faecalis*, the most common single species present in teeth after failed root canal therapy, often possesses acquired tetracycline resistance. In these cases, root canal infections are commonly treated with Ledermix^®^ paste, which contains demeclocycline, or the new alternative endodontic paste Odontopaste, which contains clindamycin; however, these treatments are often ineffective. We studied the killing activity of the cyclic antimicrobial peptide gramicidin S (GS) against planktonic and biofilm cells of tetracycline-resistant clinical isolates of *E. faecalis*. The high therapeutic potential of GS for the topical treatment of problematic teeth is based on the rapid bactericidal effect toward the biofilm-forming, tetracycline-resistant *E. faecalis*. GS reduces the cell number of planktonic cells within 20–40 min at a concentration of 40–80 μg/mL. It kills the cells of pre-grown biofilms at concentrations of 100–200 μg/mL, such that no re-growth is possible. The translocation of the peptide into the cell interior and its complexation with intracellular nucleotides, including the alarmon ppGpp, can explain its anti-biofilm effect. The successful treatment of persistently infected root canals of two volunteers confirms the high effectiveness of GS. The broad GS activity towards resistant, biofilm-forming *E. faecalis* suggests its applications for approval in root canal medication.

## 1. Introduction

Teeth with necrotic pulps and periapical radiolucencies indicate the presence of bacteria in a root canal system. They can cause apical periodontitis, an inflammatory process around the apex of a tooth root accompanied by bone resorption [1,2]. Root canal infections are characterized by a wide variety of combinations of a few oral anaerobic bacteria, such as *Actinomyces* species and peptostreptococci. The non-oral but environmental bacterium *Pseudomonas aeruginosa* is frequently isolated from treatment-resistant cases because of its intrinsic multidrug resistance, including resistance to tetracycline [3]. However, more and more evidence has been provided on the prevalence of *Enterococcus faecalis* in teeth associated with a failed endodontic treatment of root canal infections [2,4,5,6,7]. The success rate for the re-treatment of teeth with *E. faecalis* is lower (66%) than the overall success rate of re-treatment (75%) [8]. Previous studies reported about up to 77% prevalence of *E. faecalis* in teeth with failed endodontic treatment [7]. *E. faecalis* can best adapt to and tolerate the conditions in the filled root canal [6]. This facultative anaerobe is able to grow at 6.5% NaCl and temperatures up to 45 °C and is even able to colonize dentine under alkaline pH and glucose starvation [9]. It has a remarkable ability to avoid leukocyte-dependent antibacterial mechanisms and to utilize collagen within dentin, using this property for adhesion within the root canal [2]. A number of other virulence factors in *E. faecalis*, such as a plasmid-encoded surface localized adhesion protein, gelatinase (metalloendopeptidase), a serine protease, hemolysin, which is classified as a type A lantibiotic, and extracellular superoxide, permit adherence and facilitate invasion [6]. The surface adherence of biofilms is significant in persistent endodontic infections because it increases the difficulty of eliminating biofilms by conventional treatment measures [10]. Although the conventional endodontic irrigant sodium hypochlorite (1%–6%) exhibits more efficient elimination of *E. faecalis* biofilms in comparison to chlorhexidine, calcium hydroxide, povidone iodine and ethylendiamintetraacetat [11,12], this intracanal medication was found to have a limited action against bacterial biofilms [13].

Currently, there are no clinically available compounds that disassemble biofilms of *E. faecalis*, although several antimicrobial peptides are in development, such as siamycin I, which affects quorum sensing, as well as protegrin, and oritavancin that can damage the bacterial membrane [14]. Recently, the use of the commercially available Ledermix^®^ paste, which contains demeclocycline, or the new alternative endodontic paste Odontopaste, which contains clindamycin, was reported to be ineffective in the treatment of *E. faecalis* infections due to the natural clindamycin and acquired tetracycline resistance of these bacteria [6,15]. Their dominance in nosocomial infections is based on their ability to develop multidrug resistance via the acquisition of antibiotic resistance genes on plasmids or transposons from other bacteria. Notably, surface aggregation facilitates the contact between the cells, which leads to the exchange of plasmids that carry resistance. Approximately 70% of *E. faecalis* isolates from primary endodontic infections showed a resistance to tetracycline [16]. In genomic analysis, the tetracycline resistance genes *tetM* and *tetL*, as well as gelatinase, aggregation substance and enterococcal surface protein genes, were detected in these *E. faecalis* isolates.

In this paper, we report the antimicrobial and anti-biofilm activity of the cyclic decapeptide gramicidin S (GS) against tetracycline-resistant *E. faecalis* illustrated by two cases of successful treatment of Ledermix^®^-resistant root canal infections using this peptide.

## 2. Results

### 2.1. Susceptibility of Tetracycline-Resistant E. faecalis to GS

**Figure 1:**
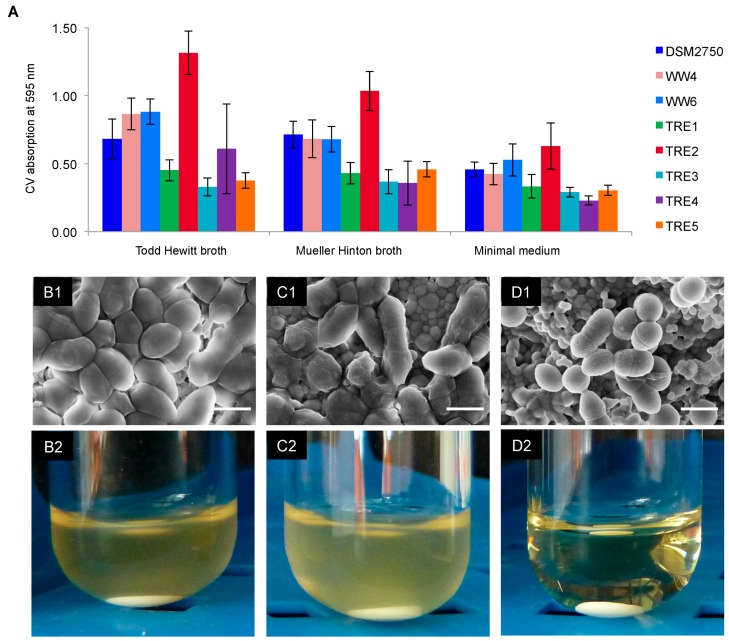
Anti-biofilm activity of GS. (**A**) When eight *E. faecalis* strains were compared using a crystal violet staining assay, the tetracycline-resistant clinical isolate TRE2 exhibited the strongest biofilm formation in all three nutrient media; (**B1**) SEM image of a TRE2 biofilm formed on a hydroxyapatite disk, which served as a control and as a starting point for antibiotic treatment; (**B2**) when B1 was incubated in TH broth, the biofilm is found to re-grow in a suspended form; (**C1**) SEM image of B1 after treatment with 400 μg/mL demeclocycline, and (**C2**) re-growth of a biofilm in suspension; (**D1**) B1 after treatment with 400 μg/mL GS; and (**D2**) no biofilm re-growth was observed after treatment, even when only 200 μg/mL of GS were used in D1. Reproducible results were obtained in two independent experiments. The scale on the SEM images is 1 μm.

According to the data of the Clinical and Laboratory Standards Institute [17], enterococci are defined to be resistant to tetracycline if the minimum inhibitory concentration (MIC) values are ≥16 μg/mL and they are called susceptible at MIC ≤ 4 μg/mL. Table 1 shows the resistance of the *E. faecalis* DSM 2570, which was isolated from urine and represents a quality control strain for antimicrobial susceptibility testing [18], the WW4 cheese isolate and four tetracycline-resistant clinical isolates, designated TRE1–TRE5. For comparison, the clinical root canal isolate WW6, which is susceptible to tetracycline (MIC < 1 μg/mL) but forms robust biofilms (Figure 1A), was also studied (Table 1). Tetracycline inhibits protein synthesis, and its action is therefore bacteriostatic. GS is a bactericidal antibiotic peptide. As shown in Table 1, its MIC, minimum bactericidal concentration (MBC), and minimum biofilm inhibitory concentration (MBIC_90_) are mostly in the range of 8–16 μg/mL for all studied *E. faecalis* strains. This small difference between inhibitory and bactericidal concentrations as well as the concentrations required for the inhibition of biofilm formation, explains the great bactericidal activity of GS, which gives bacterial cells no chance to remain alive or to respond to the stress caused by GS treatment.

### 2.2. Time-Dependent Killing Effect of GS

**Figure 2:**
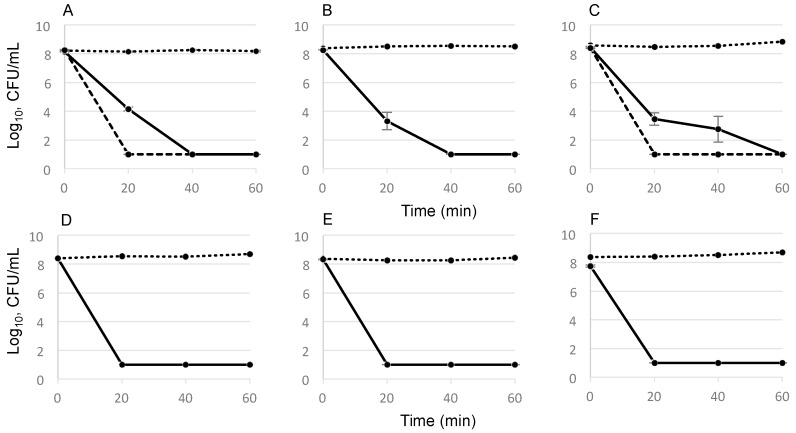
Reduction of the number of *E. faecalis* bacteria during exposure to GS: (**A**) control strain DSM 2570; (**B**) cheese isolate WW4; (**C**) root canal isolate WW6; and (**D**–**F**) clinical tetracycline-resistant isolates TRE1, TRE2, and TRE4, respectively. The bacterial number remained unchanged in the controls (dotted lines), but decreased drastically during exposure to 5 × MIC of GS (solid lines). The dashed lines show the faster bactericidal effect of 10 × MIC for DSM 2570 and WW6 strains; 10 CFU/mL was taken as the detection limit although no colonies were grown from undiluted sample. The standard deviations were calculated from at least two independent experiments.

The bactericidal activity of GS against *E. faecalis* strains over time was studied in killing assays. For this experiment, we used stationary cultures, which contained metabolically inactive, slow- or non-dividing dormant cells that tolerate conventional antibiotics and cause persistent infections [19]. At the time points of 20 min and 40 min after the addition of GS, the undiluted 100-μL bacterial aliquots revealed no colonies on agar plates. This indicates a prompt and complete reduction of the cell number in planktonic suspension, which contained 10^8^ CFU/mL cells (Figure 2). Notably, the metabolically inactive dormant cells were also killed at the studied concentrations, which corresponded to the 5 × MIC and 10 × MIC values (Table 1). The increase from 5 to 10 × MICs led to a decrease in killing time from 40 to 20 min for the *E. faecalis* DSM 2570 and WW6. The same killing curve was observed at 5 × MIC values of GS for the best biofilm former, the tetracycline-resistant clinical isolate *E. faecalis* TRE2. However, its effective concentration was 80 μg/mL because its MIC value was 16 μg/mL.

### 2.3. Biofilm-Killing Effect of GS

For these experiments, it was necessary to determine the biofilm forming capacity of all *E. faecalis* isolates to choose the strongest biofilm formers and the most suitable medium. A standard crystal violet staining assay was applied to evaluate the mass of the biofilms that were pre-grown for 24 h in 96-well microtiter plates (Figure 1A).

The strongest biofilm growth was found to occur in the Todd Hewitt (TH) broth, which contains glucose. The Mueller Hinton (MH) broth and minimal medium were less suitable for biofilm formation. The biofilm-forming capacity of the control strain DSM 2570 was comparable with that of the tetracycline-resistant WW4 (cheese isolate) and the tetracycline-susceptible WW6 (root canal isolate) (Table 1). The tetracycline-resistant clinical isolate *E. faecalis* TRE2 was the best biofilm former. Scanning electron microscopy (SEM) showed that the cells in this biofilm completely covered the surface of the hydroxyapatite disc (HAD) and were attached to the HAD nanoparticles (Figure 1B1). After exposure to 400 μg/mL of GS for 18 h, the cell morphology of *E. faecalis* TRE2 was drastically altered (Figure 1D1) in comparison to the control or to the treatment with 400 μg/mL of demeclocycline (Figure 1C1). The GS-treated cells decreased in size from 1 to 0.6–0.8 μm, the cell surface got wrinkled, and the cells detached from the HAD particles (Figure 1D1). Treatment of the pre-grown biofilms with 400 μg/mL of GS led to a disappearance of the matrix layer and to a partial elimination of biofilm cells, so that the nanoparticles of the HAD were clearly exposed underneath the cells. No re-growth of the robust TRE2 biofilm in the fresh TH broth was observed, even when only 200 μg/mL GS has been applied (Figure 1D2). This concentration represents the minimal biofilm bactericidal concentration (MBBC) of GS against *E. faecalis* TRE2, because subsequent plating of this non re-grown culture on nutrient agar did not reveal any viable cells. The control biofilm (Figure 1B2) as well as the biofilm after demeclocycline treatment (Figure 1C2) were clearly found to re-grow in the fresh TH nutrient broth after cultivation at 37 °C for 24 h. The second strongest biofilm former, the tetracycline-susceptible root canal isolate *E. faecalis* WW6 (Figure 1A), did not exhibit re-growth after treatment with 400 μg/mL demeclocycline, as expected due to its high susceptibility (see Table 1). We note, however, that the MBBC of GS against this strain was even lower (100 μg/mL) (data not shown).

### 2.4. GS Penetration into the Bacterial Cells and Binding Affinity to Nucleotides

**Figure 3:**
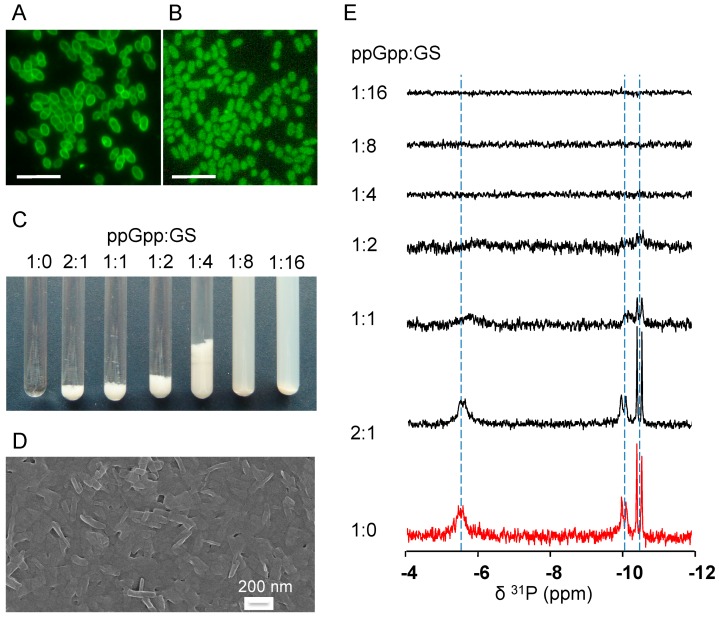
Translocation of GS into the cells and its binding affinity to the bacterial alarmon ppGpp. (**A**) Green fluorescence of the *E. faecalis* cell envelope upon staining with the dye CFSE, which cannot penetrate the cellular membrane. (**B**) Fluorescence throughout the interior of the cells is seen upon staining with a fluorescent GS-sw(FP) analog, confirming that the peptide can translocate across the cell membrane into the cytoplasm. (**C**) Co-precipitation of GS and ppGpp is seen to occur at roughly equimolar ratios, and a stable opalescent suspension is formed when GS is in large (i.e., electrostatic) excess. (**D**) SEM showed that the aggregation ppGpp by GS led to the formation of short nano-rods of about 50 nm width and 200 nm length. (**E**) When GS was added to ppGpp at different molar ratios, the ^31^P-NMR signals of the latter disappeared successively. The pure ppGpp sample (1:0) is shown in red. The scale on the fluorescent images is 5 μm.

Microbiological assays helped to elucidate the complex action of GS at the cellular level. To understand the anti-biofilm activity at the molecular level, we first studied the membrane-penetrating ability of GS, in order to find out whether it has any access to intracellular targets. For comparative fluorescent microscopy, we used the dye 5(6)-carboxy-fluorescein-*N*-hydroxysuccinimide ester (CFSE) as a control, which cannot penetrate the cell membrane. To detect GS by fluorecscence microscopy, the photo-switchable GS analog GS-sw(FP) was used, which possesses an intrinsic green fluorescence upon irradiation with visible light [20]. The fluorescence image of *E. faecalis* TRE2 stained with CFSE showed strong fluorescence of only the cell envelope (Figure 3A). On the other hand, bacteria that were incubated with GS peptide for 30 min (Figure 3B) showed an even fluorescence internally. The same result was obtained by staining the other *E. faecalis* strains (data not shown).

The access of cationic GS to the cytoplasm suggests that it may engage in electrostatic interactions with intracellular anionic targets. A key target could be the alarmon ppGpp, which regulates biofilm formation [21]. Indeed, the addition of ppGpp to GS at different molar ratios led to their co-precipitation as a result of mutual binding. At ppGpp:GS ratios of 2:1, 1:1 and 1:2 (Figure 3C), floating white flakes could be seen, suggesting the presence of large aggregates. A further excess of GS led to the formation of a stable milky opalescent suspension, which contained smaller nano-rods (Figure 3D). ^31^P-NMR analysis of the intrinsic ppGpp phosphate signals confirmed the binding and aggregation, as sharp signals were observed only in the presence of low concentrations of GS (Figure 3E). The broadening and disappearance of the ^31^P-NMR signals with excess peptide indicated the precipitation of the alarmone/GS complex. Electron microscopy of the milky suspension at a molar ratio of ppGpp:GS 1:8 (Figure 3D) revealed the assembly of GS with ppGpp into nano-rods with 50 nm width and 200 nm length. The ability of GS to bind to ppGpp and thus to deplete its free cytoplasmic pool may explain the strong inhibition of biofilm formation by GS described above (see MBIC_90_ values in Table 1).

Notably, ppGpp is not the only possible intracellular target of GS. In the literature, complex formation of GS with nucleic acids and adenosine phosphates has already been described [22,23]. Because the triphosphates GTP and ATP are utilized in the biosynthesis of ppGpp, their binding to GS may further enhance the anti-biofilm effect of the peptide. Using the same ^31^P-NMR approach as described above, we also compared the ability of several other phosphorus-containing nucleotides to bind GS. Judged by the molar ratio at which the ^31^P-NMR signal disappeared (see Figures in Supplementary Materials), the studied nucleotides exhibited GS binding affinities in the following order: ADP < ppGpp < ATP = GTP < GDP. In particular, this means that the ppGpp precursors represent even stronger complex partners for GS, and their binding to GS should additionally contribute to the overall inhibitory and bactericidal effects in bacterial biofilms.

### 2.5. Medication Reports

Case I of root canal infection: Molar 38 of a 59-year-old female patient (M.B.) was percussion-sensitive and responded negative to cold testing with CO_2_ snow. The initial root canal treatment was carried out in a standard way using irrigation with sodium hypochlorite. An intracanal Ledermix^®^ Paste dressing was administered. For the next six weeks, the tooth remained percussion sensitive and painful, so the root canals were again treated in the same way for the next four weeks, but the second treatment with Ledermix^®^ Paste was also unsuccessful. The pain was stronger and radiated into the ear and head. At the patient’s request, the tooth was medicated using a mixture of the antimicrobial peptides GS and polymyxin B (PMB) at a molar ratio of 2:1. The powders were co-dissolved in a drop of 50% ethanol and applied as an intracanal dressing with a temporary Cavit™ restoration. Alleviation of pain was experienced after one hour, and a positive healing effect was noted. After a few weeks without symptoms the tooth was sealed, and in the following five years no recurrence was observed.

**Figure 4:**
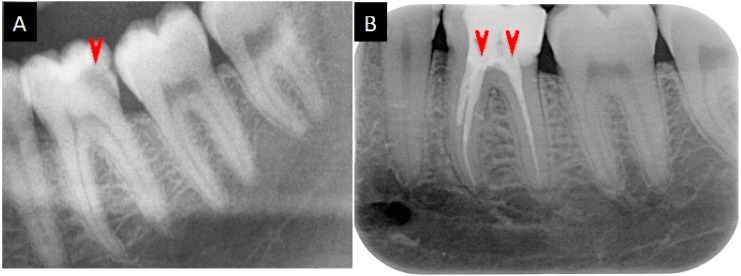
Molar 36 of a 25-year-old male patient before and after treatment. (**A**) The radiolucent area in the X-ray image indicates decay. (**B**) The sealed root canals after treatment with GS. The red arrowheads mark the decayed area and the sealed root canals, respectively.

Case II of root canal infection: Decay in molar 36 of a 25-year-old male patient (M.B.’s son) was observed by X-ray as a radiolucent spot in the pulp region (Figure 4A). Without treatment, an irreversible pulpitis developed within six months. The root canals were treated in a standard way, but a painful root canal infection appeared after two months. After the direct application of ~2 mg GS, which was suspended in sterile isotonic saline, the pain receded within an hour, and the root canals were sealed (Figure 4B). After treatment, no reoccurrence of root canal infection was observed in the following five years.

## 3. Discussion

The cyclic decapeptide gramicidin S (S = ”Soviet”) was isolated by Gause and Brazhnikova from soil bacilli and described in *Nature* and *Lancet* in 1944 [24,25]. The symmetric structure of GS is a double-repeated sequence of five amino acids, which are arranged in an antiparallel cyclic manner (_cyclo_[phe-Pro-Val-Orn-Leu]_2_) [26]. GS has an amphiphilic structure, consisting of the cationic non-canonical amino acid ornithine and the hydrophobic *^D^*phenylalanine, valine and leucine. As pharmaceuticals, antimicrobial peptides generally exhibit some unfavorable properties, such as instability, salt sensitivity, high cost of production, and a rather non-specific spectrum of activity, including hemolytic effects [27]. GS, on the other hand, is a very stable peptide. Its cyclic structure and the presence of the unusual amino acids ornithine and *^D^*phenylalanine avoid the typical proteolytic degradation by common proteases. Overall, GS has a high hemolytic activity, which limits its use to topical applications, though the addition of polyethylene glycol has been shown to prevent its hemolytic effects [28]. The production of GS via the fermentation of producing *Aneurinibacillus migulanus* phenotypes [29] is less costly than the usual chemical synthesis of antimicrobial peptides of non-bacterial origin. GS is approved as the bioactive agent in Grammidin^®^Neo, a lozenge used against sore throat and mouth ulcers, produced by Russian JSC Valenta Pharmaceuticals. Notably, despite a long treatment history, no clinical cases of bacterial resistance against GS have been reported [30].

The promising observation that GS does not lead to bacterial resistance can be attributed to its ability to attack multiple targets in bacterial cells at the same time. The first target of GS is the prokaryotic plasma membrane; therefore, the peptide possesses enhanced activity against Gram-positive bacteria like *Staphylococcus* spp., *Streptococcus* spp., and *Enterococcus* spp. [31]. In addition to depolarization, presumably via the formation of short-lived pores at sub-MICs [32], it also inhibits the respiratory enzymes NADH dehydrogenase and cytochrome *bd* terminal oxidase in the bacterial membrane [33], and it detaches several vital peripheral membrane proteins, namely the cell-division regulator MinD, the Lipid II biosynthesis protein MurG, and cytochrome c [34]. Here, we have shown that GS does not only interact with the bacterial membrane, which causes structural and functional damage as described above. In fact, it can also penetrate into the cytoplasm, where it can bind to ppGpp, the intracellular regulator of biofilm growth [35], and to its precursors ATP and GTP, as well as the energy metabolites ADP and GDP (Figures S1–S4 in the Supplementary Materials). These new modes of activity broaden the spectrum of effects known so far for GS. It is especially important to realize that this multifaceted activity profile provides GS with unique bactericidal properties toward Gram-positive bacteria irrespective of their physiological state, virulence, resistance, tolerance and phenotypic variation. Generally, the stationary phase of a liquid bacterial culture contains a population of metabolically inactive non- or slow-growing reversible phenotypes, which are known as persister cells. They are tolerant to conventional antibiotics, even at 100 × MIC [36], because these antibiotics target only growing cells. The ability of GS to disrupt membrane function and to form complexes with intracellular targets after its rapid penetration into the cell interior leads to a complete and rapid killing. This was demonstrated in our killing assays of stationary cells (Figure 2), and in the biofilm re-growth experiments with each of the selected three strongest biofilm forming strains of *E. faecalis* (Table 1 and Figure 1).

The origin of *E. faecalis* in the root canal is unclear, as enterococci do not belong to the normal oral microbial flora [7]. However, 77% of British-produced cheeses [37], 60% of French soft cheeses, and 20% of mozzarella, feta and Swiss Tilsiter cheese [38], contain enterococci. A genetic analysis of saliva, previously treated root canal samples, and of cheeses, using repetitive extragenic palindromic (REP)-PCR, revealed nine *E. faecalis* genotypes in all specimens studied. The transitional colonization of the oral cavity after the consumption of cheese may thus be a possible reason for the prevalence of *E. faecalis* in root canal infections [37,38]. Tetracycline-resistant enterococcal or multispecies infections may provoke treatment failure, requiring tooth extraction, if the therapy using an intracanal Ledermix^®^ paste dressing is applied [16]. Even if several bacteria cause the infection, a GS-containing dressing would be an unsurpassable choice for successful treatment due to the broad-spectrum activity of GS [30]. Although microbiological analysis was not performed in two above described medication cases, a cure using GS alone and in combination with PMB has been successfully achieved. Recently, we also reported that these two peptides exert a synergistic effect against multidrug-resistant strains and against biofilms of *P. aeruginosa* [39]. In the treatment of root canal infections caused by this pathogen, which is frequently isolated from treatment-resistant cases, we expect that the combination of GS/PMB could play a very promising role. Remarkably, the pain-alleviating effect, which we describe here for both medication cases, had been previously observed in Russian clinical trials as a characteristic property only of GS but not of the related tyrothricin complex [40]—a peptide mixture of the lineal gramicidin A and cyclic tyrocidines [41]. This unique property of GS should thus be highly advantageous in the treatment of painful root canal infections.

Biofilm formation is a key virulence factor in the pathogenicity of persistent *E. faecalis* infections [42]. Nutrients such as hemin, vitamin K and glucose enhance biofilm formation. The tetracycline-resistant *E. faecalis* strain TRE2 was the best biofilm former amongst all clinical isolates. The exposure of its pre-grown biofilm to an aqueous solution of GS showed a bactericidal effect (no re-growth of biofilms was observed, and no viable cells were found in this culture upon plating), and some drastic morphological alterations of the biofilm remnants were observed (Figure 3). Our results thus highlight the promising potential of GS in the successful treatment of root canal infections caused by tetracycline-resistant biofilms of *E. faecalis*. In persistent infections, these bacteria can penetrate the cellular and tissue barrier and thereby present an imminent risk to human health, causing further non-oral, life-threatening infections, such as bacteremia or endocarditis [6,43]. Therefore, the successful treatment of root canal infections and their prevention are vital tasks. GS alone or in combination with PMB offers an opportunity to help patients with untreatable root canal infections, to save their natural teeth and to avoid high prosthetic costs.

## 4. Materials and Methods

### 4.1. E. faecalis Strains, Antibiotics and the Determination of the Minimum Inhibitory Concentrations

The control strain *Enterococcus faecalis* DSM 2570 (ATCC 29212) was purchased from the German Collection of Microorganisms and Cell Cultures (DSMZ). The WW4 isolate from cheese and the root canal isolate WW6 were a kind gift from William G. Wade (Department of Microbiology, King’s College London, London, UK). The tetracycline-resistant clinical isolates TRE1-TRE5 were obtained from the medical laboratory of Staber & Kollegen, Heilbronn, Germany.

GS was produced by fermentation, extracted from producer cells and HPLC purified as described earlier [28]. Other antibiotics (tetracycline and demeclocycline hydrochlorides) were purchased form Sigma-Aldrich Chemie GmbH (Taufkirchen, Germany).

The bacterial strains were maintained at −80 °C using a Cryobank™System (Mast Diagnostica, Reinfeld, Germany). To refresh the bacterial cells, single beads were recovered in 10 mL BHI broth (Becton, Dickinson and Co., Sparks, MD, USA) in an overnight incubation at 37 °C and 200 rpm, in the 50 mL culture flasks. Single colonies were obtained by streaking these cultures on BHI agar plates, which were then stored at 4 °C. Subsequently, the colonies were used to inoculate 10 mL MH broth (Becton, Dickinson and Co., Sparks, MD, USA) to an optical density (OD) of 0.02 at 550 nm, and the cultures were grown overnight. The test cultures were prepared by inoculating 10 mL MH broth to an OD_550_ of 0.2 with the overnight cultures and allowing them to grow until the bacteria reached OD_550_ of 1–2 at 37 °C and 200 rpm. For inoculation, the test cultures were diluted immediately prior to the experiment in MH broth to obtain final inoculation doses of 5 × 10^5^ CFU/mL according to the CLSI recommendations [17].

For the determination of the MIC, the standard broth microdilution procedure [18] was modified to obtain the uniform medium concentration after the addition of antibiotic stock solutions dissolved in sterile deionized water (tetracycline and demeclocycline) or in 50% ethanol (GS). Briefly, 50 μL of the double-strength MH broth was added to the upper row of the 96-well microtiter plates (Nunclon™, Nunc GmbH & Co., Wiesbaden, Germany). Next, 50 μL of the standard MH broth was added to the remaining wells, including the columns for the ethanol control and the positive (without peptides) and negative (sterility) controls. Addition of the antibiotic stock solutions (50 μL) to the upper wells provided a standard medium concentration and the antibiotic concentration to establish a concentration gradient. Once the two-fold dilution series of the antibiotic concentration was prepared according to [44], the plates were inoculated with 50 μL of bacterial suspensions from the exponentially growing culture to reach 5 × 10^5^ CFU/mL. The plates were incubated for 22 h at 37 °C and 5% CO_2_ without agitation. To examine bacterial growth, 20 μL of an aqueous 80 μM solution of the redox indicator resazurin was added to each well. The plates were incubated for another 2 h at 37 °C. The respiration activity was calculated for each well as the difference in the absorbance of resorufin at 570 nm and resazurin at 600 nm using the microtiter plate reader FlashScan550 (Analytic Jena GmbH, Jena, Germany) and the WinFlash program. Positive values indicated bacterial growth and allowed the determination of the lowest peptide concentrations that inhibit bacterial growth. All results were obtained from several independent experiments, and each was performed in triplicate.

### 4.2. Determination of the Minimum Bactericidal Concentration (MBC)

Determination of MBC was carried out directly after the MIC assay. The 10-μL samples of all 8 dilution rows of microtiter plates were spotted on the square agar plates and incubated overnight at 37 °C and 5% CO_2_. The MBC was determined as the lowest concentration at which no bacterial growth was observed on the two parallel spotted plates in several independent experiments.

### 4.3. Determination of the Minimum Biofilm Inhibitory Concentration (MBIC)

The MBIC leading to a 90% decrease in biofilm growth (MBIC_90_) was obtained using the microdilution procedure in the same way as for the determination of MIC, but the inoculation of the wells was performed with bacterial cells from stationary cultures to reach 5 × 10^7^ CFU/mL. In contrast to the determination of MBIC for the peptide IDR-1018 in [21], we used TH broth as a medium, which facilitated maximum biofilm growth (Figure 1A). After incubation at 37 °C and 5% CO_2_ without agitation for 24 h, the planktonic cells were washed and the crystal violet staining method was applied to evaluate the biofilm growth [45] under the different peptide concentrations. The results were obtained from two independent experiments, and each was performed in triplicate.

### 4.4. Determination of the Biofilm-Forming Capacity of the E. faecalis Strains

Three nutrition media—TH broth, MH broth and minimal medium [21], containing 62 mM potassium phosphate (pH 7.0), 7 mM ammonium sulfate, 2 mM MgSO_4_, 10 μM FeSO_4_, 0.4% glucose and 0.5% casamino acids—were used to determine the best biofilm formers and the medium that supported the best biofilm growth. The central 6 × 3 wells separated by the empty columns in the 96-well microtiter plates were filled with overnight bacterial suspensions of OD_550_ = 0.2 in three different media and incubated at 37 °C and 5% CO_2_ without agitation for 24 h. The adherent biofilms were washed, dried, fixed in methanol and dried again. The staining was carried out according to previously described methods [45] in 100 μL of the 0.1% crystal violet solution for 20 min. The excess dye was removed with water, and the wells were thoroughly dried. The dye absorbed by biofilms was dissolved in absolute ethanol, and its absorption was evaluated in a microtiter plate reader at 595 nm.

### 4.5. Determination of GS Killing Activity

The killing activity of GS was monitored during the 60 min after exposure to GS concentrations of 5 × MIC and 10 × MIC if needed. The stationary cultures were diluted up to OD_550_ = 0.2 calculated for 1.5 mL culture in MH broth, including the addition of GS stock solution. The 200-μL aliquots were removed from the cultures, which were incubated in 4 mL culture tubes at 37 °C with agitation of about 220 rpm, at 0 min, 20 min, 40 min and 60 min. For each time point, the culture samples were diluted 1:10 in 900 μL MH broth and 100 μL of undiluted culture, and each dilution was spotted as ten 10 μL on the agar plates according to the drop plate method [46]. After the incubation of the plates, the bacterial growth was evaluated by the enumeration of colonies. The decrease in bacterial number indicated the killing activity for the each time point.

### 4.6. Biofilms on HAD: Scanning Electron Microscopy and Re-Growth

The three best biofilm formers were grown on HAD (3D Biotek, NJ, USA), which served as a tooth-like material. The discs were placed into the 24-well microtiter plates (Nunc GmbH & Co., Wiesbaden, Germany) and inoculated with a cell suspension from the overnight stationary cultures, which were grown in TH broth and diluted to OD_550_ = 0.2. After 30 h growth at 37 °C without agitation, biofilms on HAD were placed into solutions containing 50 μg/mL, 100 μg/mL, 200 μg/mL and 400 μg/mL of GS, 400 μg/mL of demeclocycline, or 150 mM sodium phosphate buffer (SPB, pH 7.2) for 18 h. After that, the biofilms on the HAD were washed in SPB and placed into 1 mL of fresh TH broth to examine their re-growth. Cultures were incubated at 37 °C with agitation at 200 rpm for 24 h. To determine the number of viable cells after exposure to GS, the 100 μL of TH culture after re-growth experiment were spotted as described in 4.5. The second series of biofilms on HAD after the treatment were washed in SPB, fixed for 1 h in 2% glutaraldehyde dissolved in SPB, washed twice in distilled water and dried in the sample box. The samples were sputtered to obtain a 1-nm platinum layer using the high vacuum coating system Leica EM MED020 (Leica Microsystems, Wetzlar, Germany). The biofilm images were obtained with a Supra 55 VP scanning electron microscope (Carl Zeiss, Ostfildern, Germany).

### 4.7. Fluorescence Microscopy

The fluorescence of the photo-switchable analog GS-sw(FP), described previously [20], was used to study GS translocation into the bacterial cytoplasm. Approximately 1 mL of the *E. faecalis* cell suspension adjusted to OD_550_ = 1.0 was co-incubated with 100 μg/mL of GS-sw(FP) in less active closed-ring form and incubated at 37 °C for 30 min without agitation. To observe the fluorescence this form was converted to the fluorescent open-ring form upon the irradiation with visible light directly under the microscope for 5 min. The CFSE dye (Sigma-Aldrich, St. Louis, MO, USA), which cannot penetrate the cell membrane, was applied for comparison. The bacterial suspension for this staining was resuspended in the fresh 150 mM NaHCO_3_ buffer (pH 8.3). This was necessary because at this pH, the amino groups of membrane proteins remain deprotonated and can better bind to CFSE. The dye concentration used for the cell staining was 1 μL/mL (stock solution 10 mg/mL in DMSO). The staining procedure was carried out at the same conditions (37 °C, 30 min, without agitation). The fluorescence was observed using a Axioskop 40 light microscope (Carl Zeiss Light Microscopy, Göttingen, Germany) equipped with an “A-Plan” objective (100x/1.25 Ph3), a fluorescence filter (type 09, λ_ex_ 450-490, λ_em_ 515) and a digital camera (PowerShot G5, Canon, Tokyo, Japan). The sensitivity setting ISO 50 was used in experiments with the highly fluorescent CFSE dye. Due to the low fluorescence quantum yield of GS-sw(FP), the sensitivity setting was increased to the ISO 400.

### 4.8. ^31^P-NMR Spectroscopy of GS with Nucleotides in An Aqueous Environment

The ppGpp solution was purchased from TriLink BioTechnologies (San Diego, CA, USA), and the nucleotides ATP, ADP, GTP, and GDP were purchased from Sigma-Aldrich (Munich, Germany). 600 μL of 1 mM or 0.5 mM ppGpp solution in D_2_O (pH 7.2) were added to lyophilized aliquots of GS, yielding 0.5 mM, 1.0 mM, 2.0 mM, 4.0 mM and 8.0 mM for the end volume of 600 μL and thoroughly vortexed. Each NMR sample was then allowed to remain without any perturbation at ambient temperature for at least 2 h to allow precipitate formation. Proton-decoupled ^31^P-NMR spectra were acquired on a Bruker AVANCE 400 MHz spectrometer (Bruker-Biospin, Rheinstetten, Germany), operating at a ^31^P frequency of 161.974 MHz. The experiments were performed without temperature control (room temperature) using a Bruker 5 mm BB-PABBO probe. A single pulse (30 degrees, 15 μs) using a standard pulse sequence zgpg30 (Bruker library) was used. For ^1^H decoupling, a waltz16 decoupling sequence (Bruker library) was used. A total of 3500 scans were accumulated for each spectrum with an interpulse delay of 2 s. Spectra were processed with TopSpin 3.1 software (Bruker) using line broadening of 5 Hz.

### 4.9. Medication of the Root Canal Infections

In Case I, lyophilized powders of 9.0 mg GS (1141.4 g/mol) and 5.5 mg PMB-sulfate (1385.6 g/mol) were placed onto a glass slide, mechanically mixed with a spatula, and suspended by addition a 50 μL droplet of 50% ethanol. In Case II, about 4 mg of GS powder were suspended by addition a 50 μL droplet of sterile isotonic saline. A fraction of the resulting slurry was applied to the open root canal as an intracanal dressing, employing standard filling instruments. The tooth was temporarily restored by Cavit™. When a few weeks without pain had passed, both teeth were permanently sealed. Both individual cases studies (of the first author and a family member) were carried out privately and with full responsibility and awareness of the risks, as the only alternative would have been to have the decayed tooth extracted. Both the participants in medication signed a “Note of patient consent”.

## 5. Conclusions

In this study, we have demonstrated the considerable therapeutic potential of GS, which can be used for the treatment of root canal infections caused by tetracycline-resistant *E. faecalis* and by any of its tenacious biofilm-forming strains. The rapid killing by GS is based not only on its membrane-perturbing activity but also on further intracellular effects. The latter seem to involve the ability of the peptide to cross the lipid bilayer and bind to anionic intracellular targets (e.g., the alarmone ppGpp, and energetic nucleotides), as demonstrated here. The persistently infected root canals of two patients have been successfully treated, exhibiting rapid pain-alleviating effects. The broad activity spectrum of GS should thus be especially beneficial if the taxonomic status of the bacterial burden in root canals cannot be rapidly identified. For multispecies infections containing multidrug-resistant *P. aeruginosa,* the combination with PMB can significantly promote the curing effect further. Therefore, we recommend the approval of GS alone and/or as a complex with PMB for the treatment of persistent tetracycline-resistant root canal infections.

## Figures and Tables

**Table 1:** Minimum inhibitory concentration (MIC), minimum bactericidal concentration (MBC) and minimum biofilm inhibitory concentration (MBIC) of GS against *E. faecalis*.

*E. faecalis* Strain	Resistance/Susceptibility (μg/mL) to		Antimicrobial Activity of GS (μg/mL)		
	Tetracycline	Demeclocycline			
	MIC ^a^	MIC	MIC	MBC	MBIC_90_
DSM 2570	16	8	8	8	4–8
TRE1	16	16	8	16	-
TRE2	16	32	16	16	8–16
TRE4	16	8	8	8	-
TRE5	16	16	8	16	-
WW4	64	16	8	16	-
WW6	<1	<1	8	16	8–16

^a^ MICs, MBCs and MBICs were determined in at least in two independent experiments, each performed in triplicate. MBIC was determined only for the three best biofilm-formers (see Figure 1A).

## Supplementary Materials

The following are available online at http://www.mdpi.com/1424-8247/9/3/56/s1, Figure S1. Binding of ATP by GS; Figure S2. Binding of ADP by GS; Figure S3. Binding of GTP by GS; Figure S4. Binding of GDP by GS.

## Abbreviations

The following abbreviations are used in this manuscript:

GS	gramicidin S
PMB	polymyxin B
MIC	minimum inhibitory concentration
MBC	minimum bactericidal concentration
MBIC_90_	minimum biofilm inhibitory concentration, 90% inhibition
MBBC	minimal biofilm bactericidal concentration
TRE	tetracycline resistant enterococci
CFU/mL	colony forming units per mL
OD	optical density
SEM	scanning electron microscopy
CFSE	5(6)-carboxy-fluorescein-*N*-hydroxysuccinimide ester
HAD	hydroxyapatite disc
NMR	nuclear magnet resonance analysis
MH	Mueller Hinton broth
TH	Todd Hewitt broth
BHI	brain heart infusion broth
MM	minimal medium
SPB	sodium phosphate buffer
