# Supplementary material for: Therapeutic Potential of Gramicidin S in the Treatment of Root Canal Infections
Source: Pharmaceuticals (Basel). 2016 Sep 7;9(3):56. doi: 10.3390/ph9030056 (PMC5039509; doi:10.3390/ph9030056)
Supplement: Supplementary file 1 [file pharmaceuticals-09-00056-s001.pdf]

Supplementary Materials: Therapeutic Potential of Gramicidin S in the Treatment of Root Canal Infections

Marina Berditsch, Hannah Lux, Oleg Babii, Sergii Afonin and Anne S. Ulrich

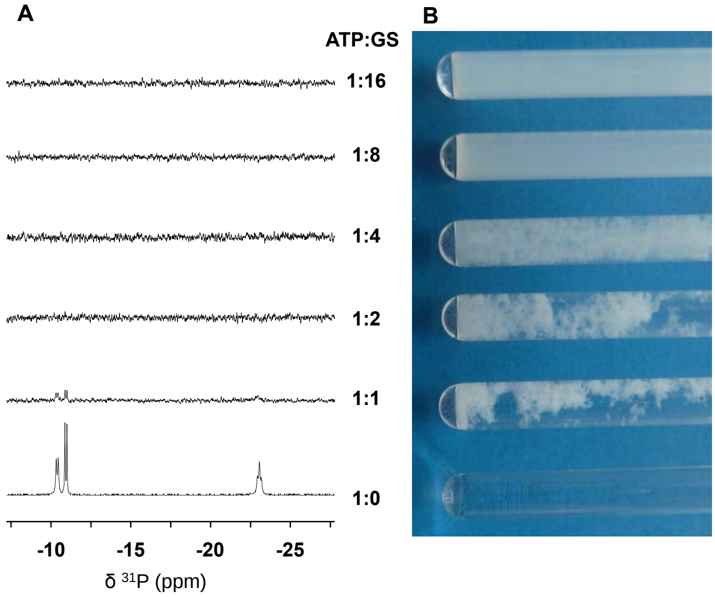

Figure S1. Binding of ATP by GS. (**A**) Upon titration, ^{31}P-NMR spectra show the complete disappearance of ATP signals already at a molar ratio of ATP:GS 1:2. (**B**) Photographs of the NMR-samples (top view) reveal the formation of various dispersions. With increasing concentration of GS, initially some large floating precipitates are observed (ATP:GS ranging 1:1 to 1:4), before the samples transform into a stable milky emulsion (above ATP:GS 1:4).

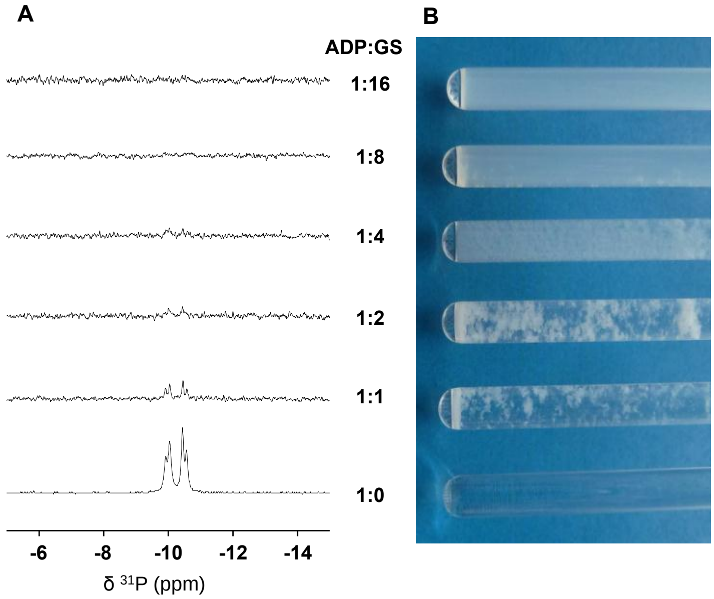

Figure S2. Binding of ADP by GS. (**A**) Upon titration, ^{31}P-NMR spectra show the complete disappearance of ADP signals already at molar ratio of ADP:GS 1:8. (**B**) Photographs of the NMR-samples (top view) reveal the formation of various dispersions. With increasing concentration of GS, initially some large floating precipitates are observed (ADP:GS ranging 1:1 to 1:4), before the samples transform into a stable milky emulsion (above ADP:GS 1:4).

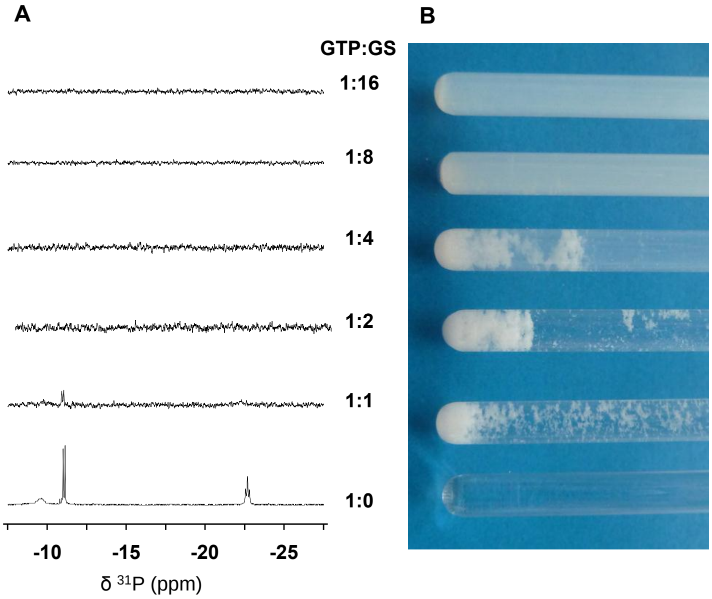

Figure S3. Binding of GTP by GS. (**A**) Upon titration, ^{31}P-NMR spectra show the complete disappearance of GTP signals already at molar ratio of GTP:GS 1:2. (**B**) Photographs of the NMR-samples (top view) reveal the formation of various dispersions. With increasing concentration of GS, initially some large floating precipitates are observed (GTP:GS ranging 1:1 to 1:4), before the samples transform into a stable milky emulsion (above GTP:GS 1:4).

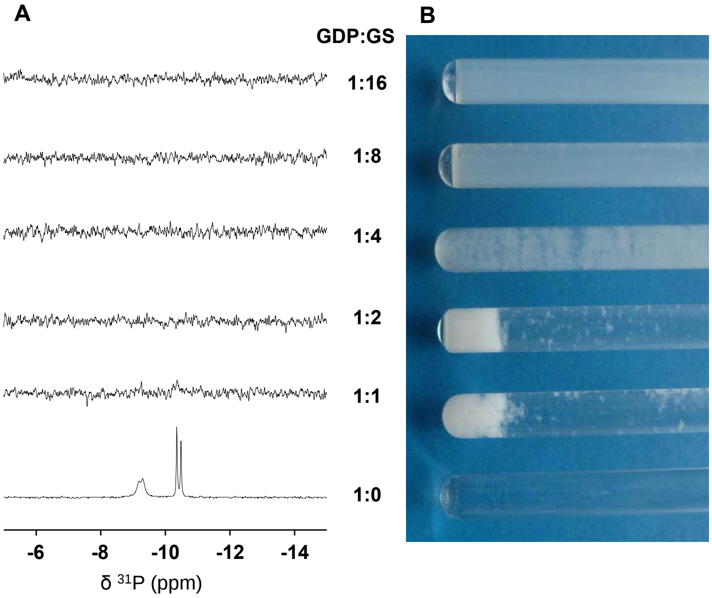

Figure S4. Binding of GDP by GS. (**A**) Upon titration, ^{31}P-NMR spectra show the complete disappearance of GDP signals already at molar ratio of GDP:GS 1:1. (**B**) Photographs of the NMR-samples (top view) reveal the formation of various dispersions. With increasing concentration of GS, initially some large floating precipitates are observed (GDP:GS ranging 1:1 to 1:4), before the samples transform into a stable milky emulsion (above GDP:GS 1:4).

© 2016 by the authors; licensee MDPI, Basel, Switzerland. This article is an open access article distributed under the terms and conditions of the Creative Commons by Attribution (CC-BY) license (http://creativecommons.org/licenses/by/4.0/).
